# Pulmonary 4D-flow MRI imaging in landrace pigs under rest and stress

**DOI:** 10.1007/s10554-024-03132-9

**Published:** 2024-05-31

**Authors:** A. Faragli, M. Hüllebrand, A. J. Berendsen, L. Tirapu Solà, F. P. Lo Muzio, C. Götze, R. Tanacli, P. Doeblin, C. Stehning, B. Schnackenburg, F. N. Van der Vosse, E. Nagel, H. Post, A. Hennemuth, A. Alogna, Sebastian Kelle

**Affiliations:** 1 Department of Cardiology, Angiology and Intensive Care Medicine, Deutsches Herzzentrum der Charité, Augustenburger Platz 1, 13353 Berlin, Germany; 2 Charité – Universitätsmedizin Berlin, corporate member of Freie Universität Berlin and Humboldt-Universität zu Berlin, Berlin, Germany; 3 Berlin Institute of Health (BIH), Berlin, Germany; 4 DZHK (German Centre for Cardiovascular Research) partner site Berlin, Berlin, Germany; 5 Deutsches Herzzentrum der Charité (DHZC), Institute of Computer-assisted Cardiovascular Medicine, Berlin, Germany; 6 Fraunhofer Institute for Digital Medicine MEVIS, Berlin, Germany; 7 Department of Biomedical Engineering, Cardiovascular Biomechanics Group, Eindhoven University of Technology, Eindhoven, The Netherlands; 8 Hospital Moises Broggi, Barcelona, Spain; 9 Department of Medicine and Surgery, University of Parma, Parma, Italy; 10 Clinical Science, Philips Healthcare, Hamburg, Germany; 11 Institute of Experimental and Translational Cardiac Imaging, DZHK Centre for Cardiovascular Imaging, Goethe University Hospital Frankfurt, Frankfurt am Main, Germany; 12 Department of Cardiology, Contilia Heart and Vessel Centre, St. Marien-Hospital Mülheim, Mülheim, Germany

**Keywords:** Cardiovascular magnetic resonance, 4D-flow MRI, Pulmonary circulation, Flow, Velocity, Pigs

## Abstract

4D-flow MRI is a promising technique for assessing vessel hemodynamics. However, its utilization is currently limited by the lack of reference values, particularly for pulmonary vessels. In this work, we have analysed flow and velocity in the pulmonary trunk (PT), left and right pulmonary arteries (LPA and RPA, respectively) in Landrace pigs at both rest and stress through the software MEVISFlow. Nine healthy Landrace pigs were acutely instrumented closed-chest and transported to the CMR facility for evaluation. After rest measurements, dobutamine was administered to achieve a 25% increase in heart rate compared to rest. 4D-flow MRI images have been analysed through MEVISFlow by two independent observers. Inter- and intra-observer reproducibility was quantified using intraclass correlation coefficient. A significant difference between rest and stress regarding flow and velocity in all the pulmonary vessels was observed. Mean flow increased 55% in PT, 75% in LPA and 40% in RPA. Mean peak velocity increased 55% in PT, 75% in LPA and 66% in RPA. A good-to-excellent reproducibility was observed in rest and stress for flow measurements in all three arteries. An excellent reproducibility for velocity was found in PT at rest and stress, a good one for LPA and RPA at rest, while poor reproducibility was found at stress. The current study showed that pulmonary flow and velocity assessed through 4D-flow MRI follow the physiological alterations during cardiac cycle and after stress induced by dobutamine. A clinical translation to assess pulmonary diseases with 4D-flow MRI under stress conditions needs investigation.

**Supplementary Information:**

The online version contains supplementary material available at 10.1007/s10554-024-03132-9.

## Introduction

While the gold standard for the assessment of the pulmonary hemodynamic is the right ventricular catheterization [1], the most reliable and most used non-invasive imaging technique for the assessment of the pulmonary vessels remains echocardiography, mainly via Doppler sonography [2, 3]. Nonetheless, common limitations such as the difficulty of obtaining a good measurement window have pointed towards exploring new imaging methods such as magnetic resonance imaging (MRI) [3, 4].

Magnetic Resonance Angiography (MRA) is an established technique employed to analyse the vascular system in clinical practice [4]. MRA is generally performed to assess the vessels’ morphology and investigate anatomical abnormalities following the administration of a contrast agent [5]. However, it is contraindicated in patients with severe renal failure and may be associated with allergic reactions, including anaphylactic shock [5, 6]. Phase-contrast MRA has emerged as a technique being able to assess both anatomy and the function of the vessels accurately by avoiding the injection of contrast agent [7]. Besides anatomy assessment, physiology details are important in making a thorough clinical diagnosis and therefore parameters such as flow amplitude and uniformity, jet velocity and regurgitant fractions are paramount additional features to characterise, stage and follow-up cardiovascular diseases. Currently, 2D phase-contrast (PC) is the most used flow-measuring image acquisition sequence utilizing velocity-encoding in a single direction. This allows the user to examine shunts, regurgitations, and collateral flows [8]. Each 2D flow measurement requires a breath-hold, making the use of this technique difficult in several patients with heart diseases of different entities [9], and may impede the consistency of the individually acquired slices. Recently, 4D-flow MRI has emerged as a promising new technique aimed at the assessment of anatomy and flow in a more accurate way than the 2D flow Nonetheless, the main challenges of 4D-flow MRI analysis encompass the post-processing of the large data obtained and the necessary corrections performed through a specific software to adjust for gradient field distortions [10]. Although the accuracy of 4D-flow MRI measurements compared to echocardiography has already been proven [11] and validation studies against standard 2D flow CMR have already been performed in humans [12], other groups have described its parameters to underestimate flow rates making further studies necessary [13, 14]. Thus, 4D-flow MRI has been mainly utilized for research purposes as the lack of software standardization and consequent reproducibility represent still an unresolved issue [15]. On the other side, there is a high need to utilize MRA techniques to develop disease models, and reference values for pre-clinical studies are lacking [16]. This is particularly true for large animals regarding pulmonary circulation, which is generally understudied compared to the aorta and arterial vessels [11, 17, 18]. A recent work by the group of Stam et al. compared invasively measured aorta flow with 2D PC-flow and 4D-flow MRI measurements in a cohort of Landrace pigs [17]. Flow measurements were performed on 4D-flow MRI images at the aortic valve level, in the ascending aorta, and the pulmonary trunk [17]. Although the authors showed a strong correlation between invasively measured hemodynamic and both 4D and 2D flow MRI, the assessment of the pulmonary vasculature was not the main purpose of the study and was not thoroughly investigated [17]. The study that mostly investigated the pulmonary vasculature in a population of large animals has been performed by the group of Roldan et al. in which they validated 4D-flow MRI determined pulmonary vascular resistance against invasive measurements in a canine cohort of acutely induced pulmonary hypertension [19]. However, the work did not provide reference values for pulse wave velocities [19].

Recently, 4D-flow MRI has emerged as a promising new technique aimed at the assessment of anatomy and flow in a more accurate way than the 2D flow Nonetheless, the main challenges of 4D-flow MRI analysis encompass the post-processing of the large data obtained and the necessary corrections performed through a specific software to adjust for gradient field distortions [10]. Although the accuracy of 4D-flow MRI measurements compared to echocardiography has already been proven [11] and validation studies against standard 2D flow CMR have already been performed in humans [12], other groups have described its parameters to underestimate flow rates making further studies necessary [13, 14]. Thus, 4D-flow MRI has been mainly utilized for research purposes as the lack of software standardization and consequent reproducibility represent still an unresolved issue [15]. On the other side, there is a high need to utilize MRA techniques to develop disease models, and reference values for pre-clinical studies are lacking [16]. This is particularly true for large animals regarding pulmonary circulation, which is generally understudied compared to the aorta and arterial vessels [11, 17, 18]. A recent work by the group of Stam etal. compared invasively measured aorta flow with 2D PC-flow and 4D-flow MRI measurements in a cohort of Landrace pigs [17]. Flow measurements were performed on 4D-flow MRI images at the aortic valve level, in the ascending aorta, and the pulmonary trunk [17]. Although the authors showed a strong correlation between invasively measured hemodynamic and both 4D and 2D flow MRI, the assessment of the pulmonary vasculature was not the main purpose of the study and was not thoroughly investigated [17]. The study that mostly investigated the pulmonary vasculature in a population of large animals has been performed by the group of Roldan et al. in which they validated 4D-flow MRI determined pulmonary vascular resistance against invasive measurements in a canine cohort of acutely induced pulmonary hypertension [19]. However, the work did not provide reference values for pulse wave velocities [19].

Imaging, and without administration of gadolinium contrast agent [20], offering several advantages compared to 2D flow [20, 21]. For instance, 4D-flow MRI analysis allows measuring velocity in all three spatial directions over time throughout the cardiac cycle (3D + time = 4D) making velocity measurements more accurate [21]. The velocity field measured with 4D-flow MRI can be used to estimate relative pressure gradients via the Navier-Stokes equation as presented in the paper by Meier et al [22]. Another advantage of the 4D-flow MRI approach is the possibility to derive time-resolved pressure maps in any vessel of interest.

Indeed, to our knowledge, no studies analysed how flow and velocity in the pulmonary trunk and pulmonary arteries are affected at rest and after dobutamine infusion, even if such stress test is generally performed in the clinics and pre-clinical studies.

Therefore, the objective of the current study was to analyse flow and velocity measured through pulmonary 4D-flow MRI under both rest and stress conditions in a cohort of Landrace pigs to provide reference values for future studies.

## Methods

The study population included n = 9 Landrace pigs selected from an already published study cohort from our group, in which dobutamine stress testing was performed [16, 23–25]. The experimental protocols were approved by the local bioethics committee of Berlin, Germany (G0138/17) and conform to the “European Convention for the Protection of Vertebrate Animals used for Experimental and other Scientific Purposes” (Council of Europe No 123, Strasbourg 1985). The range in weight of the pigs was 50.3 ± 9.2, the mean body surface area (BSA) was 2.4 ± 0.4 and no significant difference was observed between the groups.

The HR at rest was on average 104 ± 15 beats per minute (BPM), while during dobutamine infusion it reached on average 147 ± 11 BPM.

### Experimental protocol and CMR acquisition

Before being transferred to the cardiac magnetic resonance (CMR) facility, female Landrace pigs were sedated and intubated. Ventilation was support with an MRI-compatible machine (Titus, Dräger Medical, Germany) with the following standardized settings: FiO2 of 0.5, I: E-ratio of 1:1.5, positive end-expiratory pressure of 5 mmHg, and a tidal volume of 10 ml/Kg. Moreover, the respiratory rate was adjusted, when required, to maintain an end-expiratory carbon dioxide partial pressure of 35–45 mmHg. Anaesthesia was kept stable with a combination of isoflurane, fentanyl, midazolam, ketamine and pancuronium. Dobutamine infusion was titrated, aiming at a 25% heart rate (HR) increase compared to baseline values. This protocol was established by our group in a small pilot study in which titration of dobutamine was assessed by left ventricle invasive conductance measurements. In one of our previous works, we assessed the reproducibility of cardiac magnetic resonance feature tracking (CMR-FT) strain parameters in a Landrace pig cohort during different inotropic states and one of them was due to dobutamine infusion using the same threshold (25% increase in HR) [16].

MR imaging was performed on a 3T clinical MR scanner (Ingenia, Philips Healthcare). 4D PC data were acquired with a 3D T1-weighted fast field echo (FFE) sequence with flow encoding gradients in three orthogonal axes (FH, RL, AP), in combination with retrospective gating to the electrocardiograph (ECG) cycle with 25 heart phases. Data were acquired in sagittal orientation, covering the entire heart and outflow tract. An anterior- and posterior phased array coil was employed for signal reception which consists of a flexible anterior and a posterior part that is integrated into the patient bed. Up to 28 coil elements were used for signal reception, depending on the size and position of the visual field recorded.

Typical scan parameters were as follows: Acquired FOV FH/RL/AP = 180 × 87 × 288 mm³,acquired resolution = 2.8 × 2.8 × 2.8 mm³,reconstructed resolution = 1.5 × 1.5 × 2.8 mm³, velocity encoding (VENC) along all three axes = 250 cm/s, TR/TE/flip = 3.8 ms/2.4 ms/5°, SENSE acceleration factor 2, bandwidth 2500 Hz / pixel.

4D flow data were acquired during normal mechanical ventilation. Respiratory gating or motion correction were considered unnecessary as respiration-induced bulk cardiac motion was found to be minimal in the animal cohort. The scan time was on the order of 10 min. After the MRI measurements were concluded, the animals were transported back to the operating room for sacrifice.

### CMR image analysis

The resulting magnitude image and three velocities encoded images were imported in the software MEVISFlow (Fraunhofer MEVIS, Bremen, Germany) [26]. Pre-processing was applied, with noise-masking, antialiasing, automatic correction for eddy currents, and phase unwrapping as provided by MEVISFlow [27].

**Fig 1:**
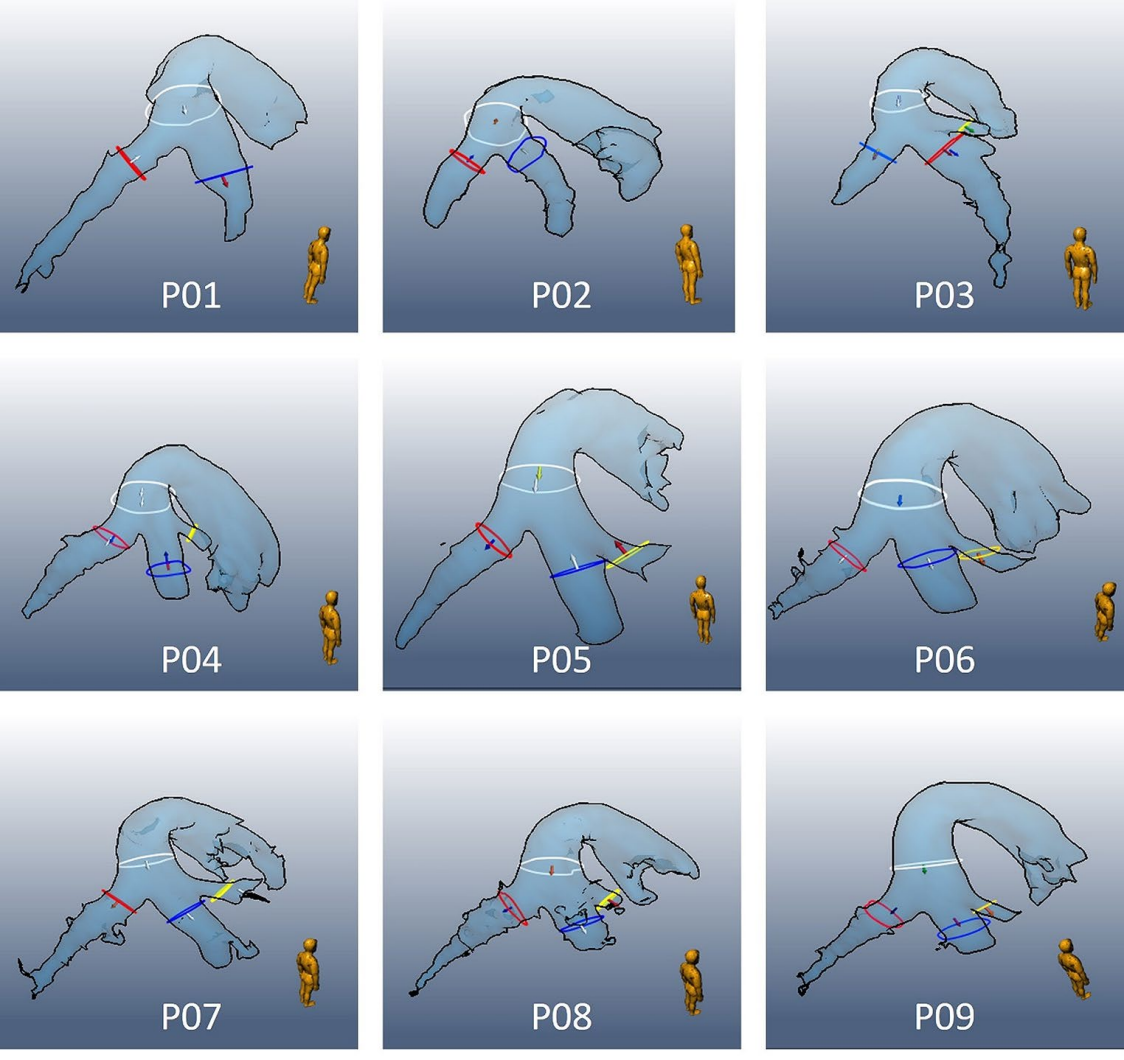
Placement of ROIs in pulmonary arteries in our pig’s cohort (n = 9). Represented in white is the main pulmonary trunk (PT), in blue the left pulmonary artery (LPA), while in red the right pulmonary artery (RPA). A proximal branch of the LPA has been detected in 7 out of 9 animals, ROI represented in yellow

It became apparent that not all imported scans started at the correct time in the cardiac cycle due to incorrect triggering on the magneto-hemodynamic (MHD) effect, particularly under stress condition. This was manually corrected by rearranging the timepoints. Afterward, the pulmonary trunk (PT) and pulmonary arteries were located and segmented semi-automatically using an interactive watershed transform on the PC-MRA, which resulted in a 3D mask. Regions of interest (ROI) were placed by manually encircling the vessel of interest at three locations (Fig. 1): in the PT, before the vessel starts to dilate, at the left pulmonary artery (LPA) just after the first branch and in the right pulmonary artery (RPA) at the same height as in the LPA. Multiplanar reformatted images (MPRI) at the same location in the TP, LPA and RPA are defined manually for rest and stress. ROIs were defined manually and were automatically transferred motion compensated to all timeframes. The propagated ROIs were manually checked for each timeframe and corrected if necessary.

The hemodynamic parameters obtained were:

- Flow: forward and backward flows.
- Regurgitant fraction.
- Mean flow.
- Peak velocity.
- Normalized Flow Displacement (NFD).

NFD is defined as the distance between the center of the flow ($\overrightarrow{{cv}_{j}}$) and the center of the vessel ($\overrightarrow{{cg}_{j}}$), normalized by the vessel diameter, following the definition by Sigovan et al. [28]. A value of 0 means the flow is centered, and 1 means the flow is not centered. NFD is described by the following formula:

$${NFD}_{j}=\frac{\left|\overrightarrow{{cv}_{j}}- \overrightarrow{{cg}_{j}}\right|}{{D}_{j}}$$ where

$$\overrightarrow{{cv}_{j}}= \frac{\sum _{i=1}^{{m}_{j}}\overrightarrow{{r}_{i}} \times \left|\overrightarrow{{u}_{i}}\right|}{\sum _{i=1}^{{m}_{j}}\left|\overrightarrow{{u}_{i}}\right|} \text{a}\text{n}\text{d} \overrightarrow{{cg}_{j}}=\frac{{\sum }_{i=1}^{{m}_{j}}\overrightarrow{{r}_{i}}}{{m}_{j}}$$

- Through Flow Degree (TFD).

TFD is defined as the average ratio of through-plane velocity magnitudes (${\overrightarrow{u}}_{TP,i}$) and the sums of through-plane $\left({\overrightarrow{u}}_{TP,i}\right)$ and in-plane (${\overrightarrow{u}}_{IP,i}$) velocity magnitude. TFD is a measure of the flow swirl, which, unlike similar parameters like helicity, incorporates vessel orientation [28]. TFD is described by the following formula:

$${TFD}_{j}= \frac{1}{{m}_{j}}\sum _{i=1}^{{m}_{j}}\frac{\left|{\overrightarrow{u}}_{TP,i}\right|}{\sqrt{{\left|{\overrightarrow{u}}_{TP,i}\right|}^{2}+{\left|{\overrightarrow{u}}_{IP,i}\right|}^{2}}}$$ where

$${\overrightarrow{u}}_{TP,i}=\left(\overrightarrow{{u}_{i}} \times \overrightarrow{{n}_{j}}\right) \overrightarrow{{ n}_{j}} \text{a}\text{n}\text{d} { \overrightarrow{u}}_{IP,i}=\overrightarrow{{u}_{i}}-{ \overrightarrow{u}}_{TP,i}$$

For evaluation of laminarity, minor flows superimposed to the main predicted flow pattern are evaluated and a value of 0 means there is no in-plane motion (no turbulence).

The angle measured in degrees was defined as the flow angulation in relation to the plane defined by the ROI area.

### Statistical analysis

Data were analysed using Microsoft Excel and IBM SPSS Statistics version 23.0 software (SPSS Inc., Chicago, IL, USA) for Windows. Figures were made with GraphPad Prism version 8. All data are presented as mean ± standard deviation (SD). The Shapiro–Wilk test was used to determine whether the data were normally distributed. Data between groups at different inotropic states were analysed by one-way ANOVA for repeated measurements. Post-hoc testing was performed by Tukey’s test. Nonparametric variables were compared using the Wilcoxon test. A p-value of < 0.05 was considered statistically significant.

### Reproducibility testing

Inter- and intra-observer reproducibility was quantified using intra-class correlation coefficient (ICC). Agreement was considered excellent for ICC > 0.74, good for ICC 0.60–0.74, fair for ICC 0.40–0.59, and poor for ICC < 0.40. Data analysis was repeated after four weeks to assess intra-observer agreement. All the operators took the measurements twice, and the average values were taken. The agreement between the measurements was further assessed with Bland-Altman analysis, investigating both intra- and inter-observer agreements [29].

## Results

Pulmonary hemodynamic parameters measured at rest and during dobutamine stress test assessed in PT, LPA, and RPA are reported in Table 1. As it can be noted a significant increase in mean flow and mean peak velocity could be observed after dobutamine infusion.

**Table 1:** Measured pulmonary hemodynamic parameters during rest and stress states

			Rest	Stress	
			Mean ± SD	Mean ± SD	p-value
PT (n = 9)		Mean flow [L/min]	0.09 ± 0.06	0.14 ± 0.05	0.004
		Reg fraction [%]	0.58 ± 0.91	0.18 ± 0.51	0.33
		Peak velocity [m/s]	0.90 ± 0.24	1.40 ± 0.21	<0.001
		Mean NFD	0.08 ± 0.02	0.09 ± 0.02	0.42
		Mean TFD	0.56 ± 0.16	0.59 ± 0.14	0.95
		Mean angle	29.98 ± 23.96	27.06 ± 22.06	0.37
LPA (n = 9)		Mean flow [L/min]	0.04 ± 0.02	0.07 ± 0.03	0.004
		Reg fraction [%]	3.47 ± 6.54	2.94 ± 4.63	0.75
		Peak velocity [m/s]	0.80 ± 0.21	1.40 ± 0.24	<0.001
		Mean NFD	0.08 ± 0.02	0.08 ± 0.02	0.25
		Mean TFD	0.56 ± 0.14	0.58 ± 0.09	0.95
		Mean angle	36.52 ± 26.02	38.78 ± 20.15	0.76
RPA (n = 9)		Mean flow [L/min]	0.05 ± 0.03	0.07 ± 0.02	0.004
		Reg fraction [%]	0.31 ± 0.51	0.24 ± 0.73	0.59
		Peak velocity [m/s]	0.80 ± 0.21	1.33 ± 0.28	<0.001
		Mean NFD	0.07 ± 0.02	0.07 ± 0.01	0.32
		Mean TFD	0.65 ± 0.06	0.62 ± 0.05	0.02
		Mean angle	16.20 ± 6.66	22.26 ± 9.76	0.01
LPA branch (n = 7)		Mean flow [L/min]	0.009 ± 0.003	0.014 ± 0.015	0.43
		Reg fraction [%]	1.438 ± 1.580	4.378 ± 6.013	0.29
		Peak velocity [m/s]	0.818 ± 0.399	1.236 ± 0.369	0.13
		Mean NFD	0.058 ± 0.019	0.043 ± 0.012	0.18
		Mean TFD	0.608 ± 0.074	0.596 ± 0.179	0.88
		Mean angle	48.78 ± 6.21	49.34 ± 19.76	0.94

The hemodynamic parameters measured during rest and stress from the pulmonary trunk (PT), the left pulmonary artery (LPA), the right pulmonary artery (RPA) and the LPA proximal Branch are displayed. Data are presented as mean ? SD. A p-value of <0.05 was considered significant. Reg Fraction = regurgitation fraction; NFD = normalized flow displacement; TFD = through flow degree

The anatomical reconstruction of the main pulmonary vessels with their ROIs of each pig are shown in Fig. 1. Moreover, a proximal secondary branch arising from the beginning of the LPA, after the bifurcation of the PT was observed in 7 out of 9 pigs (Fig. 1).

### Pulmonary 4D-flow MRI reference values in pigs at rest and stress

**Fig. 2:**
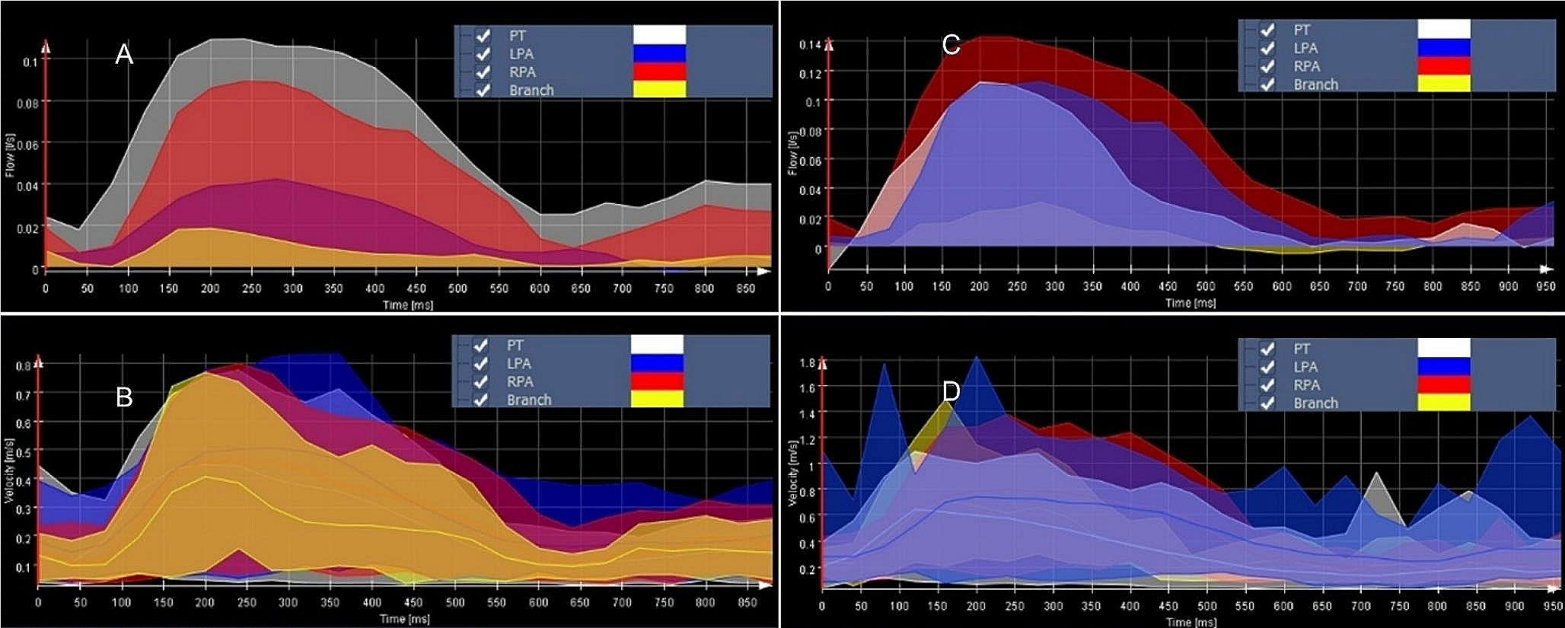
Snapshots from MEVISFlow of both flow and velocity in a representative pig during a complete myocardial cycle at rest and stress. Flow (**A**) and velocity (**B**) graphs at rest of pulmonary trunk (grey) left pulmonary artery (blue) and right pulmonary artery (red) and proximal left pulmonary artery branch (yellow) as displayed in MEVISFlow during a complete myocardial cycle. We then repeated the same analysis for flow (**C**) and velocity (**D**) at stress during dobutamine infusion

Through MEVISFlow software, it has been possible to obtain a color-coded graphical reconstruction of the respective increase and decrease of flow and velocity during systole and diastole in the PT, LPA, and RPA at both rest (Fig. 2A and 2B) and stress (Fig. 2C and 2D).

As expected, flow and velocity increase during systole and decrease during diastole. This effect is exacerbated during dobutamine stress.

**Fig. 3:**
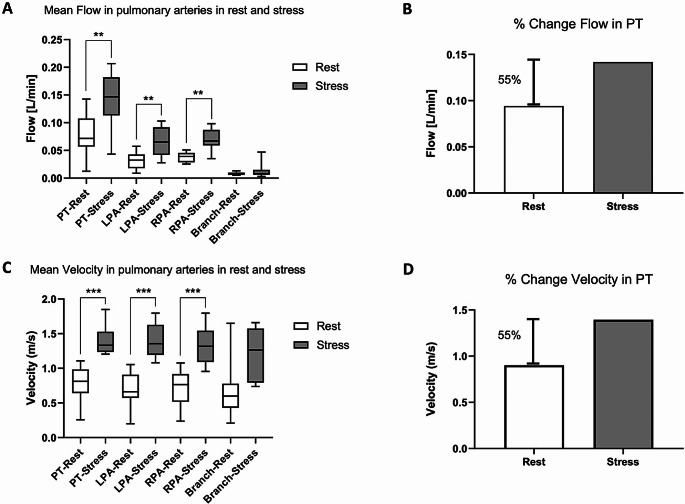
Comparison of mean flow and mean peak velocity at rest versus stress states. (**A**) Mean flows in the pulmonary arteries are plotted during rest and stress (box plot 5–95 percentile). (**B**) Mean flow percentage of change in the PT is displayed between calculated averages at rest and stress. (**C**) Mean peak velocities in the pulmonary arteries are plotted in rest and stress (box plot 5–95 percentile). (**D**) Mean peak velocity percentage of change in the PT is shown between calculated averages at rest and stress. PT = pulmonary trunk; LPA = left pulmonary artery; RPA = right pulmonary artery; Branch = proximal left pulmonary artery branch

We then represented the parameters relevant for clinical translation, namely, mean flow and mean peak velocity obtained from PT, LPA, and RPA at rest and after dobutamine administration. A significant difference has been observed for all the observed pulmonary vessels (Fig. 3A, 3 C). To highlight the sensitivity of the measurement, we calculated the relative percentage of change for both flow and velocity in PT (Fig. 3B and 3D) which was more than 50% between rest and stress. In LPA we observed an even higher increase in flow and velocity up to 75% for both parameters, while in RPA flow increased by 40% and velocity by 66%.

**Fig. 4:**
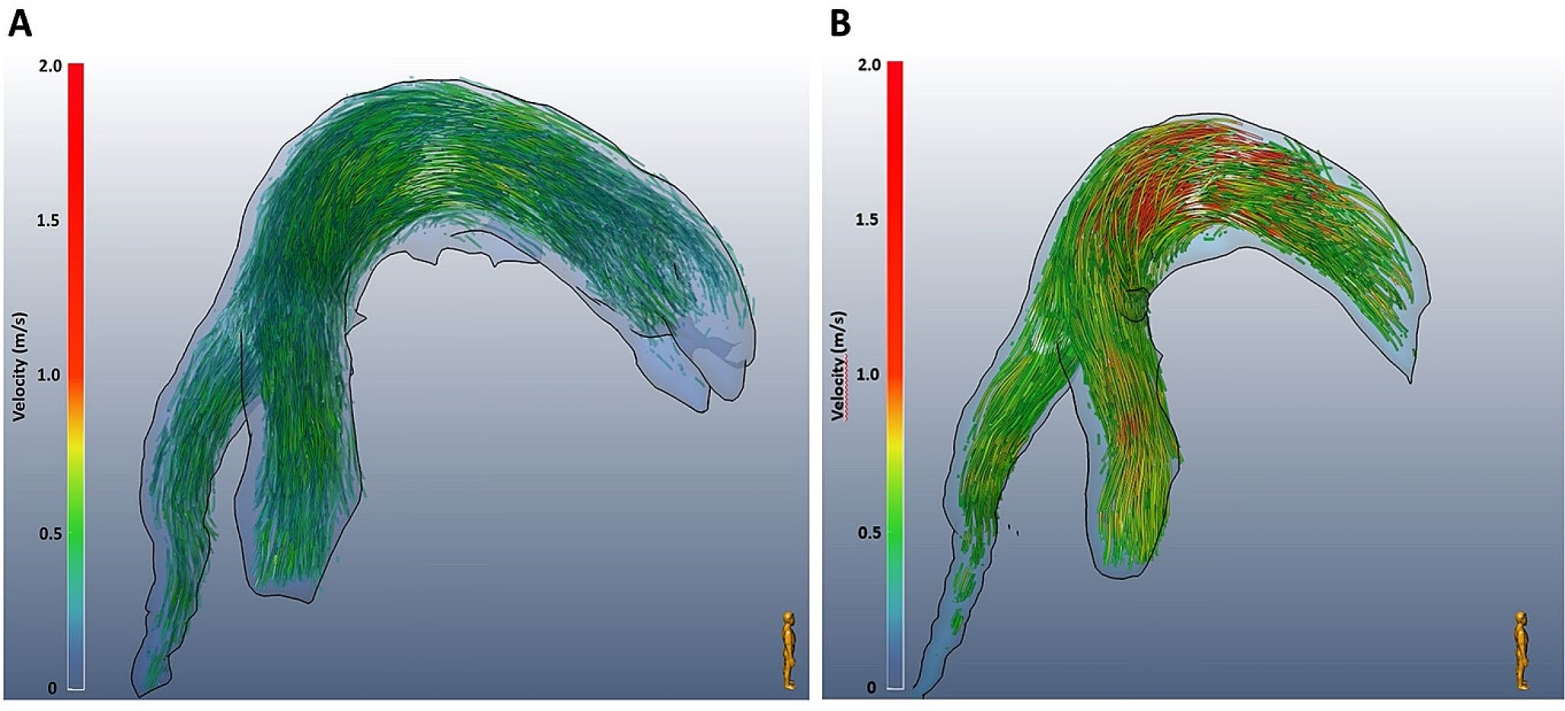
Graphical 3D reconstruction through MEVISFlow at rest versus stress. (**A**) Graphical reconstruction of velocity and flow in MEVISFlow during baseline and (**B**) after stress induced by dobutamine. The scale on the left represents velocity where red is the highest velocity compared to blue representing the lowest

Eventually, through MEVISFlow, a graphical 3D anatomical reconstruction has been obtained, analysing the flow and velocity for the whole pulmonary arterial tree at rest (Fig. 4A) and during dobutamine stress (Fig. 4B). A video of the flow reconstruction during baseline and dobutamine is presented in the Online Supplements.

### Inter and intra-observer variability

All studies were completed, and image quality was sufficient to perform the 4D-flow MRI analysis. We performed a reproducibility analysis to detect the level of agreement between observers for the measurements performed in the PT, LPA, and RPA at rest and at stress. Parameters obtained by two independent investigators, mean differences ± SD, limits of agreement, and ICC for flow and velocity parameters are presented in Tables 2 and 3. Overall, we observed a good-to-excellent (61 < ICC ≤ 99) intra- and inter-observer reproducibility at rest for both mean flow and mean peak velocity measurements for all the pulmonary vessels.

**Table 2:** Intra-observer and inter-observer reproducibility for mean flow and peak velocity during baseline measurements in the PT, LPA and RPA.

	Parameters	Mean difference±SD	Limits of agreement	ICC (95% CI)
PT				
Intra-observer variability	Mean flow	0.0002 ± 0.005	-0.01 to 0.01	0.99 (0.97–0.99)
	Peak velocity	0.01 ± 0.06	-0.11 to 0.14	0.98 (0.92–0.99)
Inter-observer variability	Mean flow	-0.0008 ± 0.01	-0.03 to 0.03	0.95 (0.77–0.98)
	Peak velocity	-0.03 ± 0.10	-0.23 to 0.17	0.96 (0.83–0.99)
LPA				
Intra-observer variability	Mean flow	0.001 ± 0.003	-0.005 to 0.007	0.98 (0.93–0.99)
	Peak velocity	-0.20 ± 0.03	-0.75 to 0.35	0.78 (0.36–0.95)
Inter-observer variability	Mean flow	0.003 ± 0.01	-0.02 to 0.03	0.69 (-0.40–0.93)
	Peak velocity	-0.01 ± 0.32	-0.78 to 0.50	0.61 (-0.70–0.91)
RPA				
Intra-observer variability	Mean flow	-0.002 ± 0.005	-0.01 to 0.009	0.90 (0.55–0.98)
	Peak velocity	-0.17 ± 0.38	-0.91 to 0.57	0.62 (-0.68–0.91)
Inter-observer variability	Mean flow	0.002 ± 0.007	-0.013 to 0.01	0.80 (0.09–0.95)
	Peak velocity	-0.02 ± 0.10	-0.23 to 0.18	0.96 (0.83–0.99)

Results are reported as mean ± SD. Agreement was considered excellent for ICC >0.74, good for ICC 0.60 – 0.74, fair for ICC 0.40 – 0.59, and poor for ICC<< 0.40. CI = confidence interval; ICC = intra-class correlation coefficient; LPA = left pulmonary artery; PT = pulmonary trunk; RPA = right pulmonary artery; SD = standard deviation

**Table 3:** Intra-observer and inter-observer reproducibility for mean flow peak velocity during dobutamine measurements in the PT, LPA, and RPA.

	Parameters	Mean difference ± SD	Limits of agreement	ICC (95% CI)
PT				
Intra-observer variability	Mean flow	0.001 ± 0.004	-0.008 to 0.01	0.99 (0.98–0.99)
	Peak velocity	-0.05 ± 0.11	-0.28 to 0.18	0.93 (0.70–0.98)
Inter-observer variability	Mean flow	0.003 ± 0.01	-0.02 to 0.03	0.99 (0.94–0.99)
	Peak velocity	-0.16 ± 0.29	-0.74 to 0.41	0.74 (-0.15–0.94)
LPA				
Intra-observer variability	Mean flow	-0.02 ± 0.03	-0.09 to 0.03	0.90 (0.58-0.98)
	Peak velocity	-0.34 ± 0.53	-1.39 to 0.70	0.24 (-2.35-0.83)
Inter-observer variability	Mean flow	0.004 ± 0.009	-0.01 to 0.02	0.96 (0.83-0.99)
	Peak velocity	-0.47 ± 0.67	-1.78 to 0.84	0.20 (-2.53-0.82)
RPA				
Intra-observer variability	Mean flow	0.0003 ± 0.007	-0.014 to 0.014	0.96 (0.82-0.99)
	Peak velocity	-0.13 ± 0.37	-0.86 to 0.60	0.24 (-0.03-0.79)
Inter-observer variability	Mean flow	-0.0002 ± 0.07	-0.01 to 0.01	0.96 (0.84-0.99)
	Peak velocity	-0.02 ± 0.40	-1.00 to 0.60	0.20 (-2.53-0.82)

Results are reported as mean ± SD. Agreement was considered excellent for ICC >0.74, good for ICC 0.60 – 0.74, fair for ICC 0.40 – 0.59, and poor for ICC< 0.40. CI = confidence interval; ICC = intra-class correlation coefficient; LPA = left pulmonary artery; PT = pulmonary trunk; RPA = right pulmonary artery; SD = standard deviation

**Fig. 5:**
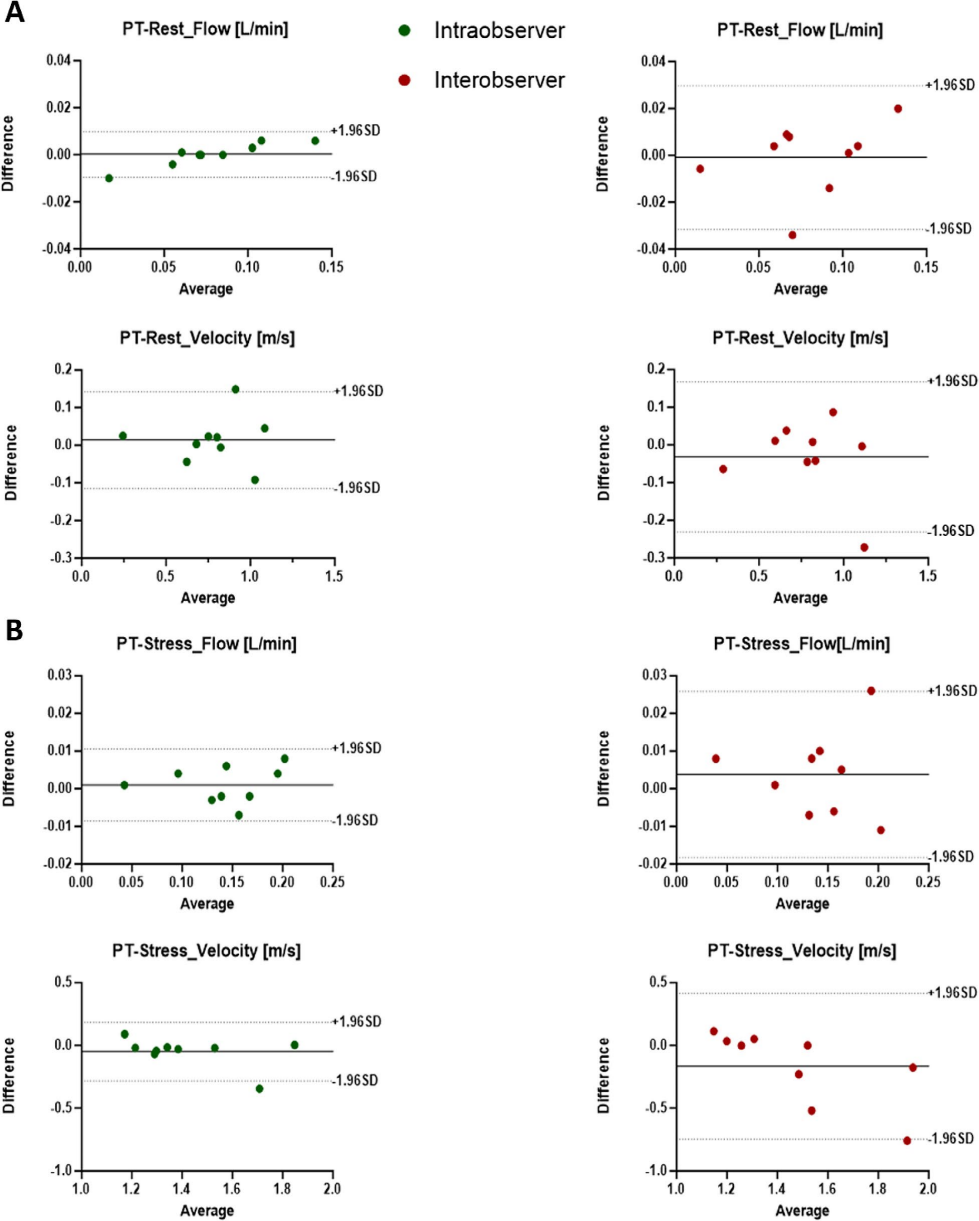
Intra- and Inter-observer reproducibility analysis of mean flow and peak velocity in the PT at rest and stress. (**A**) Upper panel: Bland-Altman plots evaluating the intra-observer reproducibility (green dots) and inter-observer reproducibility (red dots) of mean flow at rest. Bottom panel: Bland-Altman plots evaluating the intra-observer reproducibility (green dots) and inter-observer reproducibility (red dots) of mean velocity at rest. (**B**) same as A) but at stress. The average bias is represented as solid black line. The upper and lower limits of agreement (dashed black lines) are calculated as the mean difference +/- 1.96 standard deviation. PT = pulmonary trunk; SD = standard deviation

**Fig. 6:**
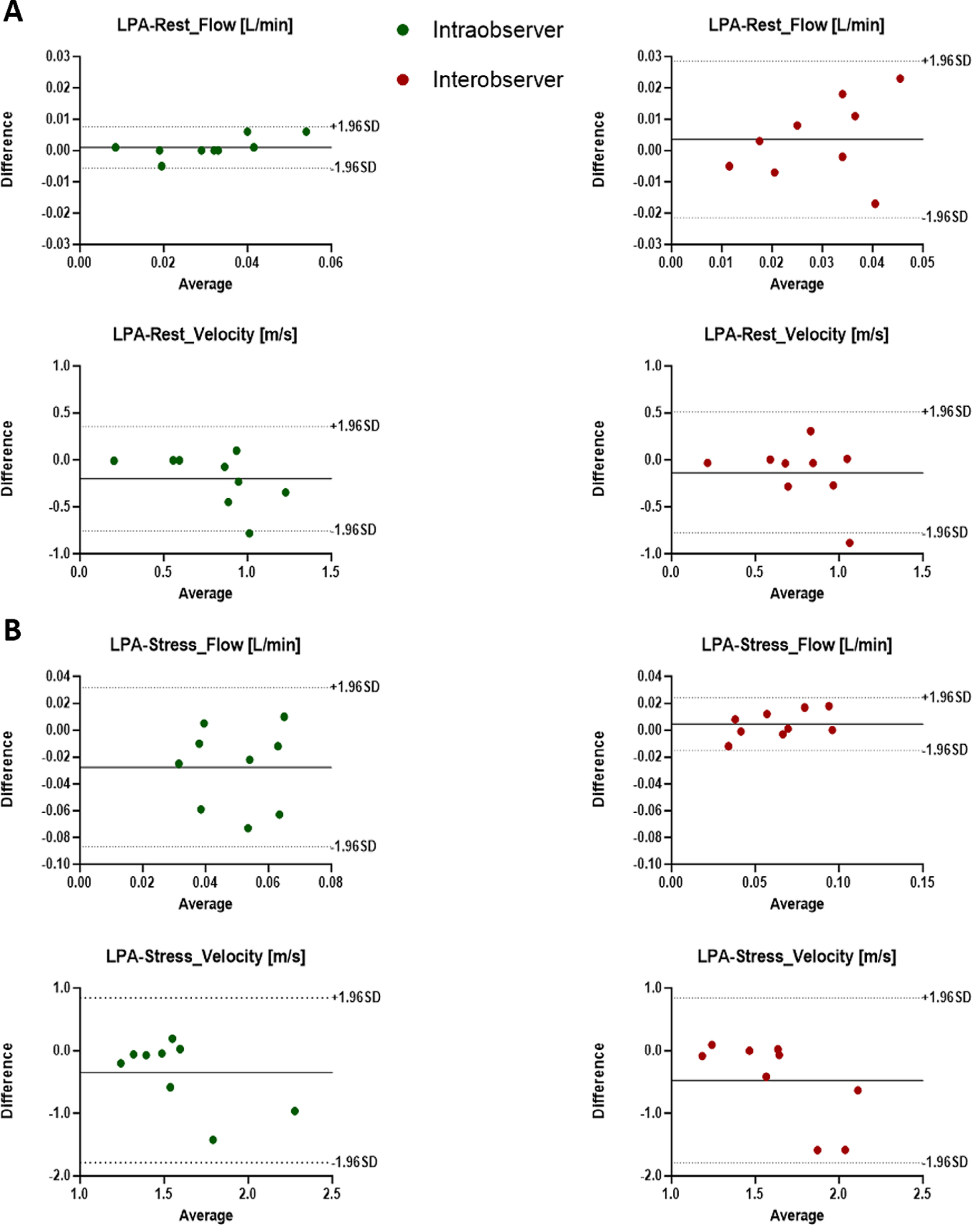
Intra- and Inter-observer reproducibility analysis of mean flow and peak velocity in the LPA at rest and stress. **A**) Upper panel: Bland-Altman plots evaluating the intra-observer reproducibility (green dots) and inter-observer reproducibility (red dots) of mean flow at rest. Bottom panel: Bland-Altman plots evaluating the intra-observer reproducibility (green dots) and inter-observer reproducibility (red dots) of mean velocity at rest. **B**) same as **A**) but at stress. The average bias is represented as solid black line. The upper and lower limits of agreement (dashed black lines) are calculated as the mean difference +/- 1.96 standard deviation. LPA = left pulmonary artery; SD = standard deviation

**Fig. 7:**
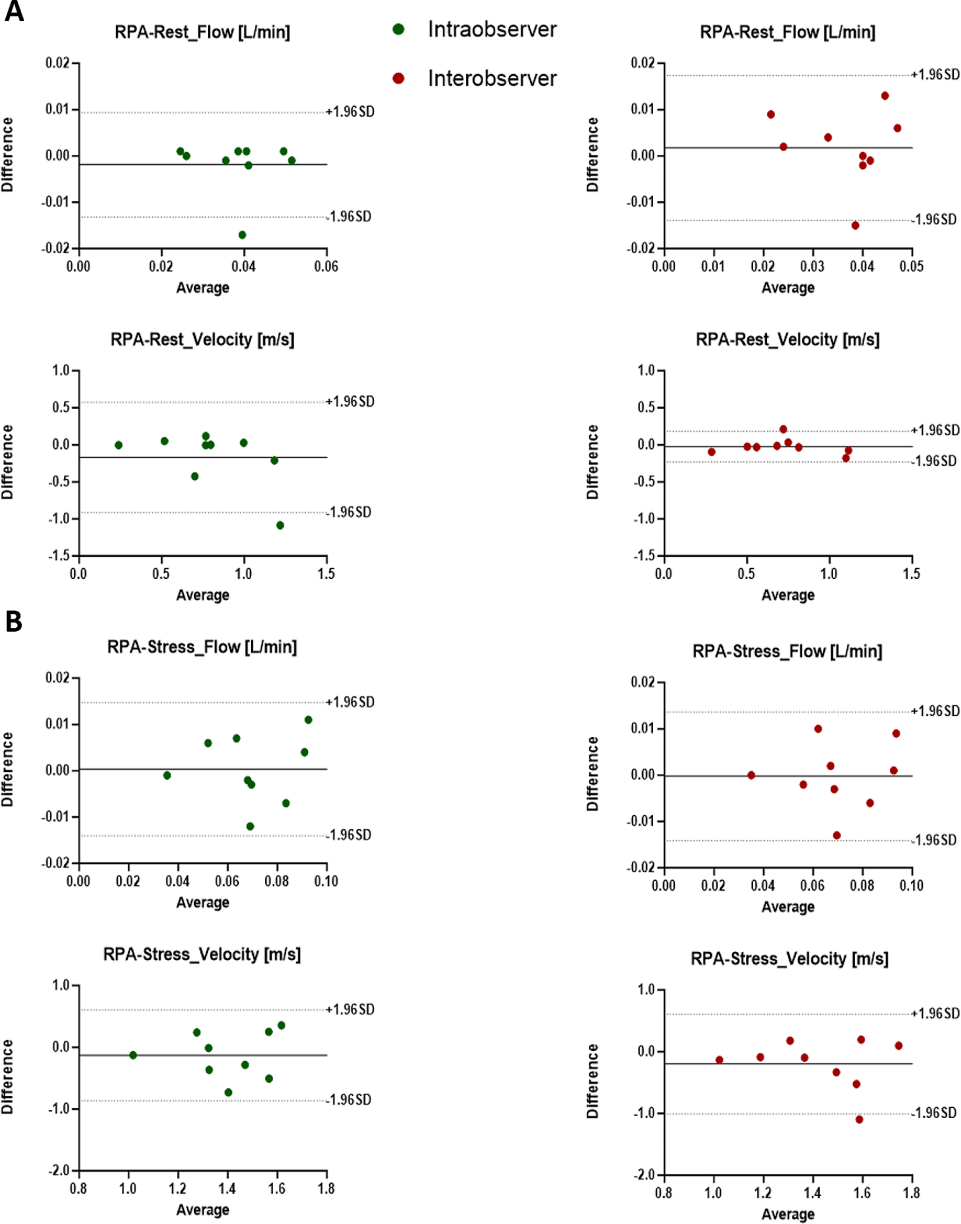
Intra- and Inter-observer reproducibility analysis of mean flow and peak velocity in the RPA at rest and stress. (**A**) Upper panel: Bland-Altman plots evaluating the intra-observer reproducibility (green dots) and inter-observer reproducibility (red dots) of mean flow at rest. Bottom panel: Bland-Altman plots evaluating the intra-observer reproducibility (green dots) and inter-observer reproducibility (red dots) of mean velocity at rest. (**B**) same as A) but at stress. The average bias is represented as solid black line. The upper and lower limits of agreement (dashed black lines) are calculated as the mean difference +/- 1.96 standard deviation. RPA = right pulmonary artery; SD = standard deviation

At stress, we observed an excellent intra- and inter-observer reproducibility for both mean flow (both ICC = 0.99) and mean peak velocity (ICC = 0.99 and ICC = 0.74, respectively) for PT measurements. Concerning LPA, we observed an excellent intra- and inter-observer reproducibility for mean flow (ICC = 0.90 and ICC = 0.96, respectively), but a poor one for mean peak velocity (ICC = 0.24 and ICC = 0.20, respectively). Eventually, we observed the same trend for RPA which showed an excellent intra- and inter-observer reproducibility for mean flow (both ICC = 0.96), but a poor one for velocity (ICC = 0.24 and ICC = 0.20, respectively). Then, we then performed a Bland-Altman Analysis to display both intra- and inter- observer reproducibility analysis, retrieving the limits of agreement presented in Tables 2 and 3 for both mean flow and mean peak velocity at rest and stress in PT, LPA and RPA (Figs. 5 and 6, and 7, respectively).

## Conclusions

The current study showed that mean flow and mean peak velocity assessed through the pulmonary 4D-flow MRI method follow the physiological alterations during systole and diastole as expected. More importantly, stress induced by dobutamine infusion increased by 40 to 60% both mean flow and velocity in all the pulmonary vessels. In conclusion, MEVISFlow can be applied for measuring pulmonary hemodynamic parameters in animals at rest and stress. Further clinical studies to assess its utility in pulmonary diseases in humans are needed.

## Discussion

The present work showed that both mean flow and peak velocity assessed in the pulmonary arteries through 4D-flow MRI finely follow the physiological alterations given by systole and diastole. Moreover, during dobutamine-induced stress, which caused a heart rate increase of 25% from baseline, we were able to observe a significant increase in both mean flow and peak velocity. As previously reported in one of our studies on the inotropic hemodynamic effects of dobutamine in pigs [23], the observed relative increase of at least 50% for both mean flow and mean velocity between baseline and stress was expected.

We did not observe an overall significant difference in the other hemodynamic parameters measured following dobutamine infusion. In detail, we analysed both NFD and TFD, as parameters of flow displacement. While some studies identified an association between increased NFD and aortic dilation [30–32] and others found it to be related to left ventricular remodelling [33], we did not observe any significant changes because our animal cohort was a healthy one. We found only a significant change in TFD after induction of stress in the RPA, however, we cannot draw any pathophysiological conclusions.

While reproducibility analysis is a standard operating procedure in clinical studies, this is not always the case in pre-clinical ones, where newly developed techniques and methodologies need strong precision and accuracy assessment. On this topic, in a previous study from our group we were able to show a high reproducibility of LV strain measured with CMR-FT [16]. In the present study, an excellent reproducibility was observed in the baseline measurements for both flow and velocity in all the pulmonary vessels.

At stress the reproducibility was found to be high for the mean flow in all the pulmonary vessels. While a good reproducibility was found also for mean peak velocity at the PT, a poor one was observed in LPA and RPA. We infer that may be due to a reduced level of accuracy of MEVISFlow in identifying the changes in the velocity vectors with decreasing vessel calibres.

The concept behind the present work is to offer reference values of healthy animals for future works, especially in large animal models where there is a need to keep the number of experiments at the minimum. This pilot study may be useful for the realization of future animal models of disease, concerning the pulmonary and right ventricle disease which remain relatively understudied.

Moreover, in the current work we were able to describe the flow and velocity of a proximal left pulmonary branch in 7 out of 9 pigs. Previous works in swine described the anatomical occurrence of up to 6 pulmonary veins [34], while another study reported the presence of a proximal right pulmonary branch [35], making our finding of a proximal additional left pulmonary branch expected. Nonetheless, we were able to report for the first time the flow and velocity analysis of such a branch unveiling the possibilities that 4D-flow MRI may offer.

Nevertheless, our study is not exempted from limitations that need to be addressed. The experiments were performed during anaesthesia, being a possible confounder for reproducibility of the measurements. The study is limited due to the small number of animals, and larger sample size may be required to detect more subtle differences. The addition of a 25% dropout rate (proportion of eligible subjects who will not complete the study or provide only partial information) before planning a study can further increase the final sample size and confirm the present results. Nonetheless, in one of our previous studies we were able to show that 5-to-11 pigs are required to achieve a 10% change in global longitudinal and circumferential strains, therefore, even if applied to other parameters, we can reasonably state that our animal cohort (n = 9) might be sufficient to evaluate major differences between groups.

Furthermore, we have employed unmodified protocols that had previously been validated in congenital heart disease and involved VENC up to 250 cm/s. In retrospect, the averaged peak velocities encountered in our animal cohort were found to be 90 cm/s and 140 cm/s during rest and stress, respectively. Therefore, smaller VENCs would have been feasible for reduced noise in the velocity maps, without the risk for wraparound artifacts in the (heterogeneous) flow patterns in the acquired 4D-flow MRI volume.

Flow datasets were checked for motion artifacts by the observers. Rest and stress images were not co-registered and the ROIs drawn at rest were not transferred to the stress dataset. The observers were trained to identify the location of MPRI manually, which might introduce additional errors.

### Electronic supplementary material

Below is the link to the electronic supplementary material.

Supplementary Material 1

## Abbreviations

MRI: Magnetic Resonance Imaging
MRA: Magnetic Resonance Angiography
PC: Phase Contrast
CMR: Cardiac Magnetic Resonance
HR: Heart Rate
CMR-FT: Cardiovascular Magnetic Resonance Feature Tracking
FFE: Fast Field Echo
ECG: Electrocardiograph
VENC: Velocity Encoding
PT: Pulmonary Trunk
ROI: Region Of Interest
LPA: Left Pulmonary Artery
RPA: Right Pulmonary Artery
MPRI: Multiplanar Reformatted Images
NFD: Normalized Flow Displacement
TFD: Through Flow Degree
SD: Standard Deviation
ICC: Intraclass Correlation Coefficient
BSA: Body Surface Area
BPM: Beats Per Minute
