# Supplementary material for: Pulmonary 4D-flow MRI imaging in landrace pigs under rest and stress
Source: Int J Cardiovasc Imaging. 2024 May 31;40(7):1511–24. doi: 10.1007/s10554-024-03132-9 (PMC11258182; doi:10.1007/s10554-024-03132-9)
Supplement: Supplementary file 1 — Supplementary Material 1 [file 10554_2024_3132_MOESM1_ESM.docx]

**Online Supplements**

**Video 1. Visual reconstruction of the flow and velocity assessed with 4D-flow MRI.** Flow and velocity assessed during rest (A) and stress induced by dobutamine (B). The grey area represents the range of interest where the measurement in the pulmonary trunk was assessed. (<https://1drv.ms/p/s!AlU4t-DkOwAPvAtUKpTylArKpaQr?e=w3w5fv>).
